# Feasibility and efficacy of 50 W ablation with the TactiFlex catheter for the initial pulmonary vein isolation of atrial fibrillation

**DOI:** 10.1002/joa3.13191

**Published:** 2024-11-22

**Authors:** Kazuhisa Matsumoto, Naomichi Tanaka, Wataru Sasaki, Tsukasa Naganuma, Masataka Narita, Daisuke Kawano, Hitoshi Mori, Kenta Tsutsui, Yoshifumi Ikeda, Takahide Arai, Shintaro Nakano, Kazuo Matsumoto, Ritsushi Kato

**Affiliations:** ^1^ Department of Cardiology Saitama Medical University, International Medical Center Hidaka Japan; ^2^ Department of Cardiology Teikyo University Itabashi Japan

**Keywords:** 50 W ablation, first‐pass isolation, impedance drop, radiofrequency ablation, TactiFlex catheter

## Abstract

**Background:**

A novel contact force (CF) sensing catheter with a mesh‐shaped irrigation tip (TactiFlexTM SE, Abbott), is expected to provide safe and effective radiofrequency ablation. Our previous study revealed that the TactiFlex catheter needs a higher power for pulmonary vein isolation (PVI) due to the long tip length. This study aimed to examine the feasibility and safety of a 50 W ablation with the TactiFlex for PVI of atrial fibrillation (AF).

**Methods:**

A PVI was performed in 100 AF patients using TactiFlex catheters with a 50 W setting, 5‐20 g CF, and 15–20 s ablation time. The primary outcomes included a successful PVI, the incidence of first‐pass isolations (FPIs), the presence of PV conduction gaps, and the incidence of complications.

**Results:**

FPIs were achieved for 82/100 (82%) right pulmonary veins (RPVs) and 87/100 (87%) left PVs (LPVs). Among the unsuccessful RPV FPIs, residual carina potentials were observed in 16/18 cases (89%), PV gaps in 1/18 cases (5.5%), and both carina and PV gaps in 1/18 cases (5.5%). Similarly, among the unsuccessful LPV FPIs, residual carina potentials were observed in 11/13 cases (84.6%), PV gaps in 1/13 cases (7.7%), and both carina and PV gaps in 1/13 cases (7.7%). Periesophageal nerve injury occurred in 1/100 cases (1%), and no cardiac tamponade occurred. The overall AF‐free rate at one‐year was 81.7%.

**Conclusions:**

The 50 W ablation with the TactiFlex demonstrated a high rate of an FPI, low incidence of PV gaps, and proved to be a safe and effective approach for the initial PVI of AF.

## Graphical abstract

The First‐pass isolation (FPI) group had a significantly greater impedance drop compared to the non‐FPI group, with cut‐off values of 13.5 ohms for the LPV and 14.5 ohms for the RPV.

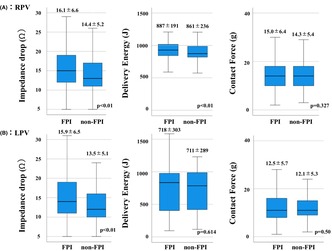

## 1 INTRODUCTION

Radiofrequency (RF) ablation has been established as the cornerstone of atrial fibrillation (AF) ablation.^1^, ^2^ Modern ablation catheters incorporate advanced irrigation technologies that serve to cool the electrode during RF ablation to prevent overheating, thereby reducing the risk of char and thrombus formation.^3^, ^4^ Previously, irrigation technologies such as the 6‐hole version (found in TactiCathTM SE, Abbott) required a high flow rate of 30 mL/min and were sometimes still inadequate for effective cooling of the ablation tip. Recently, an innovative contact force (CF) sensing catheter with a mesh‐shaped irrigation tip (“TF”, TactiFlex™ SE, Abbott) has been introduced into clinical practice.^5^, ^6^ This innovative tip design significantly reduces the irrigation flow rate from 30 mL/min to 13 mL/min while still reducing the incidence of char and thrombus formation. Although this novel catheter helps us to determine the CF information with effective cooling of the electrode, the TF catheter lacks an ablation indicator such as a lesion index or ablation index, and the effective ablation indicator of this catheter remains unknown. In our previous study, we systematically assessed the lesion formation characteristics of the TF catheter in an ex vivo experimental model.^7^ Our findings indicated that the 50 W ablation setting is necessary to create transmural lesions for pulmonary vein (PV) isolation (PVI) due to its long catheter tip. However, the practical effectiveness of a 50 W ablation with this catheter in a clinical setting remains unclear. This study aimed to investigate the efficacy and feasibility of a 50 W ablation with the TF catheter in the initial PVI of AF.

## 2 METHODS

### 2.1 Study population

One hundred consecutive AF patients who underwent a PVI with the TF catheter from September 1, 2023, to July 31, 2023, at Saitama Medical University International Medical Center were retrospectively enrolled. This study was performed in accordance with the provisions of the Declaration of Helsinki and local regulations. The research protocol was approved by the hospital's institutional review board (IRB #2023–072). In this study, the data collection was conducted using an opt‐out approach to ensure a comprehensive and unbiased sample representation. Patients who were part of the study were informed about their inclusion in the research and were given the opportunity to decline participation or withdraw their data from the study.

### 2.2 Ablation procedure

Ablation was conducted under deep sedation with a continuous infusion of propofol and dexmedetomidine with continuous monitoring of the blood pressure, oxygen saturation, and bispectral index. The intraprocedural activated clotting time was maintained between 300 and 400 s using intravenous unfractionated heparin (initial bolus 100 U/kg). The Ensite™ X System (software version 2, Abbott, Illinois, US) was used for 3D mapping in all patients. The bipolar electrocardiograms were filtered at 30–400 Hz for the electrophysiological analysis (CardioLab; GE Healthcare Japan, Tokyo).

### 2.3 Ablation settings

**FIGURE 1:**
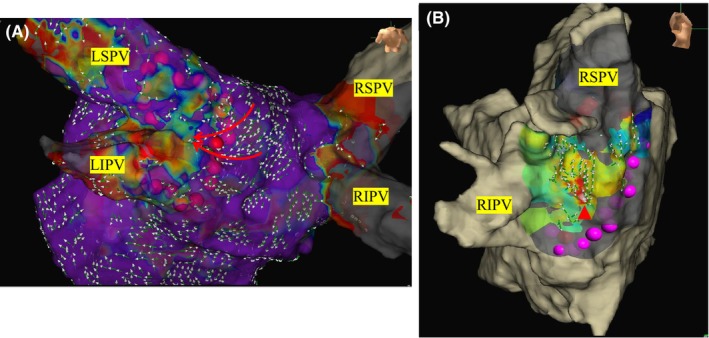
Shows the PV gap (A) and carina connection (B). The left image shows the potential conduction into the PV through a gap in the posterior wall line (yellow arrow), as indicated by the HD grid (A). The right image shows the potentials emerging centrifugally from the carina (red arrow), indicating a carina connection (B).

Venous access was established percutaneously from the right femoral vein to introduce electrode catheters into the right atrium (RA), coronary sinus, and left atrium (LA). A 6‐Fr catheter with 20 electrodes (BeeAT; Japan Lifeline, Tokyo) was inserted via the right femoral vein into the CS. After the transseptal puncture, a mapping catheter (HD Grid™ SE; Abbott, Illinois, US) was positioned in the LA. An extensive encircling PVI (EEPVI) was performed with a TF catheter using a steerable sheath (Agilis®, Abbott) by 7 different EP doctors including the 2 attending physicians and 5 EP fellows. The steerable sheath was kept in the LA and the ablation catheter was maneuvered under sheath guidance. The lesion tag size was set at 4 mm. The RF applications were delivered with a 50 W setting, CF ranging from 5 to 20 g, and duration of 15–20 s with an inter‐lesion distance of less than 5 mm. It is known that the baseline impedance affects the lesion formation.^8^ Therefore, we set the ablation time to 20 s for baseline impedance values of 120 ohms or higher, and 15 s for all other cases. An esophageal temperature probe (SensiTherm Multi®; Abbott) was inserted to prevent esophageal injury. During ablation near the esophagus on the posterior wall of the LA, the energy deliveries were limited to up to 7 s, and if the esophageal temperature exceeded 39°C, the energy delivery was halted due to concerns of esophageal injury. PVI achievement was defined as the disappearance of the electrical potentials within the PV and the confirmation of exit block by pacing from the PV. In cases in which PV potentials remained after the initial PVI, mapping was performed using an HD Grid™ SE catheter to locate the conduction gaps, and ablation was added until an electrical isolation was achieved (Figure 1). After performing the PVI, 2 μg of isoproterenol and 20 mg of adenosine triphosphate were administered intravenously to identify the presence of any dormant conduction. When dormant conduction was observed after the ATP administration, the affected sites were included as touch‐up sites and additional ablation was performed at the earliest activation site. The ATP administration was repeated until the dormant conduction was completely eliminated.

Cavo‐tricuspid isthmus ablation was performed in all cases. When ectopic beats originated from the posterior wall of the LA or the superior vena cava (SVC), a box isolation or SVC isolation was performed.

### 2.4 Study outcomes

**FIGURE 2:**
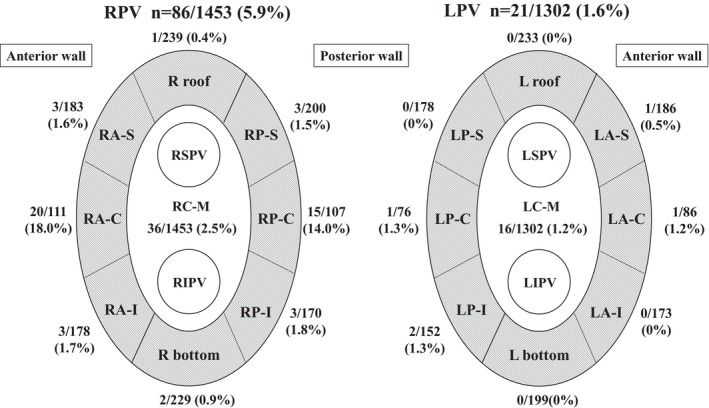
Shows the number of energy deliveries and additional applications (AP) performed at each site. In the RPVs, out of 111 ablation applications for RC‐A, 20 (18%) were additional APs, and out of 107 energy deliveries for RC‐P, 15 (14%) were additional APs. These areas required more additional ablation applications than the other parts. AP, Application, Rroof, Right roof, RA‐S, Right anterior superior, RA‐C, Right anterior carina, RA‐I, Right anterior inferior, Rbottom, Right bottom, RP‐S, Right posterior superior, RP‐C, Right posterior carina, RP‐I, Right posterior inferior, RC‐M, Right carina middle, Lroof, Left roof, LA‐S, Left anterior superior, LA‐C, Left anterior carina, LA‐I, Left anterior inferior, Lbottom, Left bottom, LP‐S, Left posterior superior, LP‐C, Left posterior carina, LP‐I, Left posterior inferior, LC‐M, Left carina middle.

Each PV was divided into nine parts (8 parts around the PVs and the carina) in order to evaluate which parts required additional RF applications (Figure 2). The incidence of a first‐pass isolation (FPI), the total ablation time, and the fluoroscopic time were evaluated as efficacy outcomes. Ablation complications such as cardiac tamponade and thromboembolic events were evaluated as safety outcomes. Furthermore, the patients underwent mandatory ECG recordings at 1, 3, 6, and 12 months and Holter ECGs at 3, and 12 months post‐PVI to evaluate the AF recurrence. We defined a recurrence as AF lasting for more than 30 s without antiarrhythmic drugs (AADs). In cases of long‐standing AF or AF recurrence within the blanking period, AADs were administered. After 3 months, the dosage of the AADs was gradually reduced, and the recurrence of arrhythmias was evaluated after the discontinuation of the AADs. For those without recurrence of AF, anticoagulants were discontinued in patients with a CHADS2 score of 1 or less and continued in those with a CHADS2 score of 2 or more.

### 2.5 Statistical analysis

The statistical analyses were performed with the Statistical Package for the Social Sciences for Windows (SPSS, version 27, Chicago, IL, USA). All variables are expressed as the mean ± SD. Statistically significant differences were identified using a one‐way analysis of variance (ANOVA) with a Tukey–Kramer post‐hoc analysis. A receiver‐operating characteristic (ROC) curve analysis and the area under the ROC curve were used to evaluate the cutoff level to predict the FPI. A value of *p* <.05 was considered statistically significant, unless specified otherwise.

## 3 RESULTS

### 3.1 Study population and baseline characteristics

A total of 100 AF patients were enrolled in this study. Table 1 shows the baseline patient characteristics. The average age was 68.3 ± 10.9 years, with males comprising 73%. The type of AF was predominantly paroxysmal AF (PAF), accounting for 61% of the cases. The mean left ventricular ejection fraction (LVEF) was within the normal range, and the left atrium diameter (LAD) was slightly enlarged (41.5 ± 7.8 mm). In 68 cases (68%), AADs (mainly β‐blockers) were administered during the perioperative period.

**TABLE 1:** Patient characteristics.

Baseline characteristics (*n* = 100)		Medical therapy	
Age (years)	68.3 ± 10.9	Antiarrhythmic drugs, *n* (%)	68 (68)
Male, n (%)	73 (73)	Oral Anticoagulant, n (%)	100 (100)
BMI (kg/m2)	24.1 ± 4.1	Laboratory data	
Paroxysmal AF, n (%)	61 (61)	Creatinine (mg/dL)	0.99 ± 0.89
CHADS2 score	1 [1–2]	NT‐Pro BNP (pg/ml)	1011 ± 1160
Chronic Heart failure, n (%)	19 (19)	Echocardiographic data	
Hypertension, n (%)	40 (40)	LVEF (%)	57.3 ± 16.3
Diabetes, n (%)	18 (18)	LAD (mm)	41.5 ± 7.8
History of Stroke, n (%)	4 (4)	LVDd (mm)	48.4 ± 6.1

*Note*: The continuous variables are shown as the mean ± SD for parametric data, median (IQR) for non‐parametric data, and the number (%) for categorical variables.

Abbreviations: AF, atrial fibrillation; BMI, body mass index; LVEF, left ventricular ejection fraction; LAD, left atrium diameter; LVDd, left ventricular diastolic diameter.

### 3.2 Procedural outcomes

Table 2 shows the procedural outcomes. A PVI was achieved in all 100 cases. An FPI was achieved for 82% of the right PVs (RPVs) and 87% for the left PVs (LPVs). In the patients an FPI of the RPVs could not be achieved, it was attributable to PV gaps in 2 cases, and a carina connection in 17 cases. In one of the two cases with a PV gap, a carina connection was also observed simultaneously. Similarly, in the LPVs, residual conduction was due to PV gaps in 2 cases and carina connections in 12. In one of the two cases with a PV gap, a carina connection was also observed simultaneously. An SVC isolation was carried out in 17% and a Box isolation in 5% of the cases. Both the procedure and fluoroscopy times included those maneuvers. Gastric hypomotility was observed in one case who underwent a Box isolation, but no fatal complications such as cardiac tamponade or atrio‐esophageal fistulae were encountered. In a case in which gastric hypomotility occurred, a PVI was performed with the same parameters as in the other cases, using an output of 50 W and CF of 10–20 g, with ablation near the esophagus limited to 7 s. That patient had persistent AF, and both the roof line and bottom line were ablated under the same settings. It was likely that the gastric hypomotility resulted from the linear ablation across the entire posterior wall of the LA, which involved ablation across the esophageal area. Gastric motility was medically restored through the cessation of oral intake and the use of gastrointestinal prokinetic agents.

**TABLE 2:** Detailed catheter ablation data.

Procedural data (*n* = 100)		PVI data	
Procedure time (min)	103 ± 29	Right PVI	
Fluoroscopy time (min)	15.5 ± 7	Number of RPV applications	1453
PVI success, *n* (%)	100 (100)	First‐pass isolation of RPV, n (%)	82 (82)
CTI ablation, *n* (%)	100 (100)	Touch up for Gap‐related site, n (%)	2 (2)
SVC isolation, *n* (%)	17 (17)	Touch up for Carina, *n* (%)	17 (17)
Box isolation, *n* (%)	5 (5)	Left PVI	
Procedural Complications, *n* (%)	1 (1)	Number of LPV applications	1302
Cardiac tamponade, *n* (%)	0 (0)	First‐pass isolation of LPV, n (%)	87 (87)
Thromboembolic events, *n* (%)	0 (0)	Touch up for Gap‐related site, n (%)	2 (2)
Gastric hypomotility, *n* (%)	1 (1)	Touch up for Carina, n (%)	12 (13)

*Note*: The continuous variables are shown as the mean ± SD for parametric data and categorical variables as the number (%).

Abbreviations: CTI, cavo‐tricuspid isthmus; LPV, left pulmonary vein; PVI, pulmonary vein isolation; RPV, right pulmonary vein; SVC, superior vena cava.

Table 3 show the differences in the procedural outcomes between the attending doctors and EP fellows (86.8 ± 34.3 vs. 107.1 ± 27.2 min, *p* =.003). The attending doctors had significantly shorter procedure times than the EP fellows. However, there was no significant difference in the ratio of an FPI (RPV; 81.3 vs. 83.3%, *p* =.839, LPV; 93.8 vs. 85.7%, *p* =.381).

**TABLE 3:** Detailed data of the differences between the attending doctors and EP fellows.

Operator (number of cases)	Attending doctors (*n* = 16)	EP fellows (*n* = 84)	*p* value
Right PVI			
First‐pass isolation of RPV, *n* (%)	13/16 (81.3)	70/84 (83.3)	.839
Touch up for Gap‐related site, *n* (%)	0 (0)	2/84 (2.4)	.533
Left PVI			
First‐pass isolation of LPV, *n* (%)	15/16 (93.8)	72/84 (85.7)	.381
Touch up for Gap‐related site, *n* (%)	0 (0)	2/84 (2.4)	.533
Procedural data			
Procedure time (min)	86.8 ± 34.3	107.1 ± 27.2	.003
Fluoroscopy time (min)	12.9 ± 7.1	16.0 ± 6.9	.12

*Note*: The continuous variables are shown as the mean ± SD for parametric data and categorical variables as the number (%).

Abbreviations: LPV, left pulmonary vein; PVI, pulmonary vein isolation; RPV, right pulmonary vein.

### 3.3 Ablation lesion analysis between the FPI group and non‐FPI group

Detailed data on the ablation lesions were available in 50 out of 100 cases. As summarized in Table 4 and Figure 2, a total of 2755 ablation lesions were analyzed (RPV 1453 lesions, LPV 1302 lesions).

**TABLE 4:** Lesion analysis.

RPV	All	R roof	RA‐S	RA‐C	RA‐I	R bottom	RP‐S	RP‐C	RP‐I	RC‐M
RF AP	1453	239	183	111	178	229	200	107	170	36
Additional AP	82	1	3	20	3	2	3	15	3	31
Delivery Energy (J)	877 ± 201	879 ± 192	901 ± 168	893 ± 127	896 ± 159	884 ± 222	861 ± 227	846 ± 172	844 ± 276	886 ± 142
RF duration (s)	18.5 ± 3.9	18.6 ± 3.6	18.9 ± 3.0	18.7 ± 2.2	18.8 ± 3.1	18.6 ± 4.5	18.2 ± 4.5	18.0 ± 3.2	18.1 ± 5.4	18.3 ± 2.9
Impedance drop (Ω)	15.6 ± 6.3	15.4 ± 5.5	14.1 ± 4.4	16.0 ± 5.8	16.3 ± 5.3	17.0 ± 8.2	15.0 ± 5.2	16.4 ± 8.0	15.1 ± 7.3	15.7 ± 4.9
Contact force (g)	14.9 ± 6.2	12.1 ± 5.1	15.3 ± 5.8	17.4 ± 5.3	16.7 ± 5.6	15.1 ± 6.9	13.2 ± 5.6	15.3 ± 6.3	16.5 ± 6.9	12.7 ± 5.1

LPV	All	L roof	LA‐S	LA‐C	LA‐I	L bottom	LP‐S	LP‐C	LP‐I	LC‐M
RF AP	1302	233	186	86	173	199	178	76	152	19
Additional AP	16	0	1	1	0	0	0	1	2	11
Delivery Energy (J)	717 ± 301	838 ± 249	871 ± 223	846 ± 235	847 ± 241	680 ± 297	571 ± 285	468 ± 252	456 ± 265	764 ± 248
RF duration (s)	15.1 ± 6.1	17.5 ± 5.0	18.2 ± 4.4	17.6 ± 4.7	17.7 ± 4.9	14.4 ± 6.0	12.2 ± 5.8	10.0 ± 5.1	9.8 ± 5.3	15.9 ± 5.1
Impedance drop (Ω)	15.6 ± 6.4	18.1 ± 7.0	15.7 ± 6.2	15.6 ± 6.1	15.4 ± 6.1	15.5 ± 6.9	14.9 ± 5.8	13.3 ± 4.0	13.8 ± 5.5	15.4 ± 6.2
Contact force (g)	12.5 ± 5.6	12.6 ± 5.8	11.4 ± 4.7	11.4 ± 5.6	11.2 ± 5.4	13.4 ± 4.8	13.0 ± 5.7	13.7 ± 6.5	12.7 ± 5.1	13.8 ± 5.0

*Note*: The continuous variables are shown as the mean ± SD for parametric data.

Abbreviations: AP, additional procedure; LA‐C, left anterior carina; LA‐I, left anterior inferior; LA‐S, left anterior superior; Lbottom, left bottom; LC‐M, left carina middle.LP‐C, left posterior carina; LP‐I, left posterior inferior; LP‐S, left posterior superior; Lroof, left roof; RA‐C, right anterior carina; RA‐I, right anterior inferior; RA‐S, right anterior superior; Rbottom, right bottom; RC‐M, right carina middle; RF, radiofrequency; RP‐C, right posterior carina; RP‐I, right posterior inferior; RP‐S, right posterior superior; Rroof, right roof.

In the RPVs, the average delivered energy was 877 ± 201 J, with an RF duration of 18.5 ± 3.9 s, impedance drop of 15.6 ± 6.3 ohms, and CF of 14.9 ± 6.2 g. In the LPVs, the average delivered energy was 717 ± 301 J, with an RF duration of 15.1 ± 6.1 s, impedance drop of 15.6 ± 6.4 ohms, and CF of 12.5 ± 5.6 g.

**FIGURE 3:**
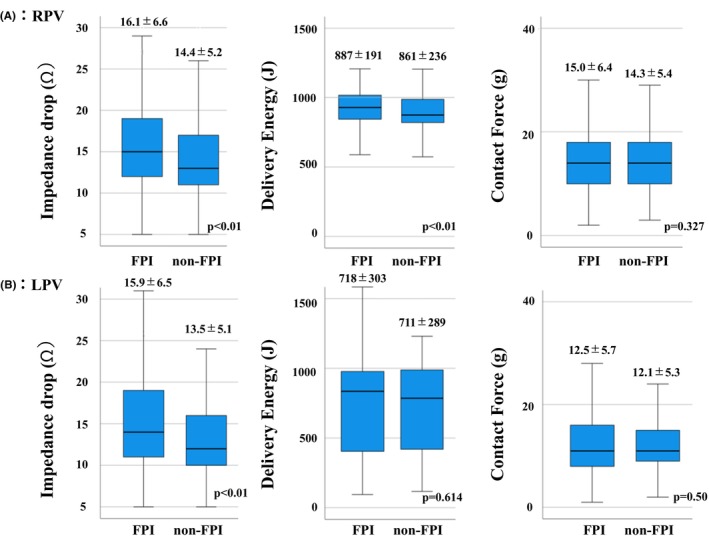
Shows the differences in the impedance drop, delivery energy, and contact force between the FPI and non‐FPI groups. In the RPVs, the impedance drop and delivery energy in the FPI group was significantly higher than that in the non‐FPI group. In the LPVs, the impedance drop in the FPI group was significantly higher than that in the non‐FPI group. FPI, First‐pass isolation.

Figure 3 shows a comparison of the impedance drop, delivered energy, and CF between the FPI and non‐FPI groups. The impedance drops were significantly greater in the FPI group for both the RPVs and LPVs (FPI group: 16.1 ± 6.6 ohm vs. non‐FPI group: 14.4 ± 5.2 ohm in RPV [*p* <.01] and FPI group: 15.9 ± 6.5 ohm vs. non‐FPI group: 13.5 ± 5.1 ohm in LPV [*p* <.01]). For the LPV, the AUC was 0.612 and the Youden index was greatest at an impedance drop of 13.5 ohms, with a sensitivity of 0.566 and specificity of 0.595 (Figure S1). For the RPV, the AUC was 0.586 and the Youden index was greatest at an impedance drop of 14.5 ohms, with a sensitivity of 0.526 and specificity of 0.621 (Figure S1).

The delivered energy was significantly higher in the RPVs in the FPI group (FPI: 887 ± 191 J vs. non‐FPI: 861 ± 236 J [*p* <.01]), however, the difference was small. There was no significant difference in the delivered energy between the groups for the LPVs. Similarly, there was no significant difference in the CF between the two groups for both the RPVs and LPVs.

### 3.4 One‐year follow‐up

**FIGURE 4:**
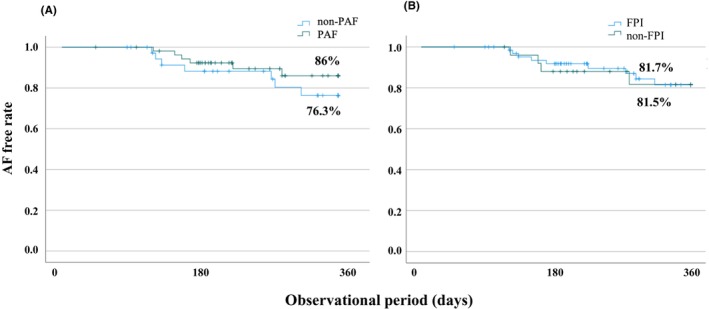
Kaplan–Meier curve of the recurrence during the 1‐year follow‐up period in the two groups (PAF, non‐PAF [A] and FPI and non‐FPI [B]). PAF, Paroxysmal atrial fibrillation, FPI, First‐pass isolation.

Ninety‐three of 100 patients were successfully followed up for one‐year (PAF: 55, non‐PAF: 38 and FPI: 67, non‐FPI: 26). The overall AF‐free rate at one‐year was 81.7%. The AF‐free rate in the PAF group was 86.0% and in the non‐PAF group 76.3%. There was no difference between the PAF and non‐PAF groups (*p* =.328) (Figure 4A). The AF‐free rate in the FPI group was 81.7% and 81.5% in the non‐FPI group. There was no significant difference between the two groups (*p* =.930) (Figure 4B).

Of the 16 AF recurrent cases, a second session was conducted in 10 cases. Among those, two cases exhibited PV line reconnections and five cases had no PV line reconnections but had residual potentials on the carina. The remaining three cases had no PV line reconnections or potentials on the carina.

## 4 DISCUSSION

### 4.1 Major findings

The major findings of our study were as follows:

1. A PVI was achieved in all 100 cases, with an FPI rate of 82% for the RPVs and 87% for the LPVs.
2. In the cases in which an FPI was not achieved, residual carina potentials were more common than PV gaps (PV gap 2/100, carina 17/100).
3. There was no significant difference in the ratio of an FPI between the attending doctors and EP fellows.
4. The FPI group had a significantly greater impedance drop.
5. No fatal complications such as tamponade were observed.
6. The PAF group had a high AF‐free rate at 1 year (86.0%).

### 4.2 Utility of a 50 W ablation for PV isolation with the TactiFlex™ SE catheter

PVs contribute to the triggers and substrate of AF^9^, ^10^ and the PVI has become the cornerstone strategy for the treatment of AF^11^, ^12^ Late reconnections caused by an insufficient lesion lead to the recurrence of AF.^13^, ^14^, ^15^ Regarding this point, the creation of transmural lesions is indispensable for AF ablation. However, a longer ablation is related to complications such as steam pops or collateral damage to the esophagu**s**.^16^ A previous study reported the procedural outcomes, safety, and non‐recurrence rates of high‐power (HP) ablation and low‐power (LP) ablation using the TF catheter.^6^ In that study, the PVI was performed with an output of 40 W‐50 W, but the RF lesion quality was left to the operator's discretion, which may have included ablation conditions insufficient for an effective PVI. As a result, the 12‐month freedom from AF recurrence in the HP group was 76.4%, which was lower than in our study. In our current study, the PVI was performed using specified ablation times based on the results of prior experiments, and as a result, the 12‐month freedom from AF recurrence was 76.3%–86.0%, which was better than in the previous study. Furthermore, the previous report did not mention the FPI. An FPI has been reported not only to reduce AF recurrence but also to have a high durability of the PVI.^17^ In our study, the 50 W ablation with the TF catheter had a high FPI rate, suggesting the possibility of a high durability of the PVI. In our study, residual conduction on the PVI line was less than 5% for each PV, suggesting that using 50 W for a 15–20 s energy delivery at each ablation site is sufficient as an ablation endpoint. The majority of cases where an FPI was not achieved were due to the carina conduction. Carina potentials have been reported to be related to epicardial connections via the septopulmonary bundle or connections with the RA (carina‐RA connections).^18^, ^19^, ^20^ The wall thickness of the PV antrum varied among the patients, and carina conduction occurred in cases with a thicker myocardium and those with epicardial connections. Our results suggested that evaluating the carina potentials could be useful in cases where an FPI is not achieved with the TF catheter ablation. Despite achieving a high rate of an FPI, no serious complications such as cardiac tamponade or strokes were observed. That may be related to the irrigation structure of the TF catheter. The TF catheter has a mesh‐shaped irrigation tip, which has been shown to reduce the risk of char and thrombus formation on the catheter. It is noteworthy that the ablation in this study was not performed by a single operator but by multiple operators including EP fellows. This study involved 2 attending doctors and 5 EP fellows. As shown in Table 3, the attending doctors had a significantly shorter procedural time compared to the EP fellows. We think that was because the attending doctors were more experienced with the procedure. However, the important point was that there was no significant difference in the ratio of an FPI between the two groups. That demonstrated the feasibility of a 50 W ablation using the TF catheter. In addition, we evaluated the rate of AF recurrence 1 year after the ablation. Although there was no significant difference between the PAF and non‐PAF groups, the AF‐free rate tended to be higher in the PAF group (86.0%). Because this study included cases with a CTI ablation and SVC isolation, and conventional follow‐up methods such as 12‐lead ECGs and Holter ECGs, the results cannot be directly compared with previous reports of AF recurrence after ablation. However, the clinical outcome appears to be favorable.

Previous report have noted that FPI is associated with a high PVI durability and reduced AF recurrence.^13^ However, there was no significant difference about the rate of AF recurrence between the FPI group and non FPI group. In our study, carina conduction was a typical reason in the non‐FPI group. The carina connection was not due to a line gap caused by insufficient ablation, but rather it appeared as a result of an epicardial connection. Additional ablation targeting the carina successfully eliminated this epicardial connection. Consequently, it is believed that there was no significant difference in outcomes between the FPI group and the non‐FPI group.

### 4.3 Relationship between the FPI and impedance drops

Our findings indicated that the FPI group exhibited a significantly greater impedance drop (Figure 3). The cut‐off values for the impedance drop required to achieve FPI were 13.5 ohms for the LPV and 14.5 ohms for the RPV. Impedance drop would become an effective parameter to predict the FPI. However, the AUC was relatively small (LPV 0.612, RPV 0.586). Furthermore, previous reports have indicated that an impedance drop of 18 ohms or more carries a risk of steam pop, and the difference is only 4–5 ohms.^21^ Further research is needed to determine whether this can be applied as an ablation indicator in clinical practice.

Additionally, since the update to version 3 of the Ensite X software, impedance drops are now represented as an ‘averaged impedance drop’. Impedance drops are affected by cardiac and respiratory oscillation. Applying the filters to remove that artifact could potentially improve the accuracy of predicting the lesion size. Whether this will become a new ablation indicator for the TF requires further study.

## 5 LIMITATIONS

There were several limitations to our study. Firstly, this study assessed the outcomes of the PVI during ablation procedures and up to one‐year of follow‐up. Therefore, how this relates to future AF recurrence rates warrants further investigation. Secondly, the procedure and fluoroscopy times did not solely reflect the PVI as additional procedures such as an SVC isolation and posterior wall isolation were performed. Thirdly, this study did not investigate the generator impedance for each patient. When the generator impedance was low, the amount of current through the tissue increased with the same output, resulting in a larger lesion size and a higher risk of steam pops. In cases where the generator impedance falls below 100 ohms, it may be advisable to refrain from delivering energy for more than 15 s. Finally, this study was retrospective and at a single center in Japanese patients. Regarding the evaluation of AF recurrence, our study evaluated AF recurrence using 24‐hour Holter monitoring. Our results might have differed if longer follow‐up devices, such as two‐week Holter monitors or implantable cardiac monitoring devices, were used.

## 6 CONCLUSION

The initial PVI of AF using a 50 W ablation with the TF catheter was effective and safe. Evaluating the carina potentials could be useful in cases where an FPI is not achieved with the TF catheter ablation.

## AUTHOR CONTRIBUTIONS

HM and RK, study conception and design; KM, WS, DK, NT, and MN, data collection and data analysis; KT, YI, TA, SN, and KM manuscript revision and study supervision.

## FUNDING INFORMATION

All the authors declare no funding information.

## CONFLICT OF INTEREST STATEMENT

All the authors declare no conflicts of interest.

## ETHICS STATEMENT

This study was performed in accordance with the provisions of the Declaration of Helsinki and local regulations. The research protocol was approved by the hospital's institutional review board (IRB #2023–072).

## CONSENT

In this study, the data collection was conducted using an opt‐out approach to ensure a comprehensive and unbiased sample representation. Patients who were part of the study were informed about their inclusion in the research and were given the opportunity to decline participation or withdraw their data from the study.

## Supporting information


Figure S1.

## Data Availability

Data not available due to the nature of this research, participants of this study did not agree for their data to be shared publicly, so supporting data is not available.
