# Supplementary figures and images for: Feasibility and efficacy of 50 W ablation with the TactiFlex catheter for the initial pulmonary vein isolation of atrial fibrillation
Source: J Arrhythm. 2024 Nov 22;41(1):e13191. doi: 10.1002/joa3.13191 (PMC11730719; doi:10.1002/joa3.13191)

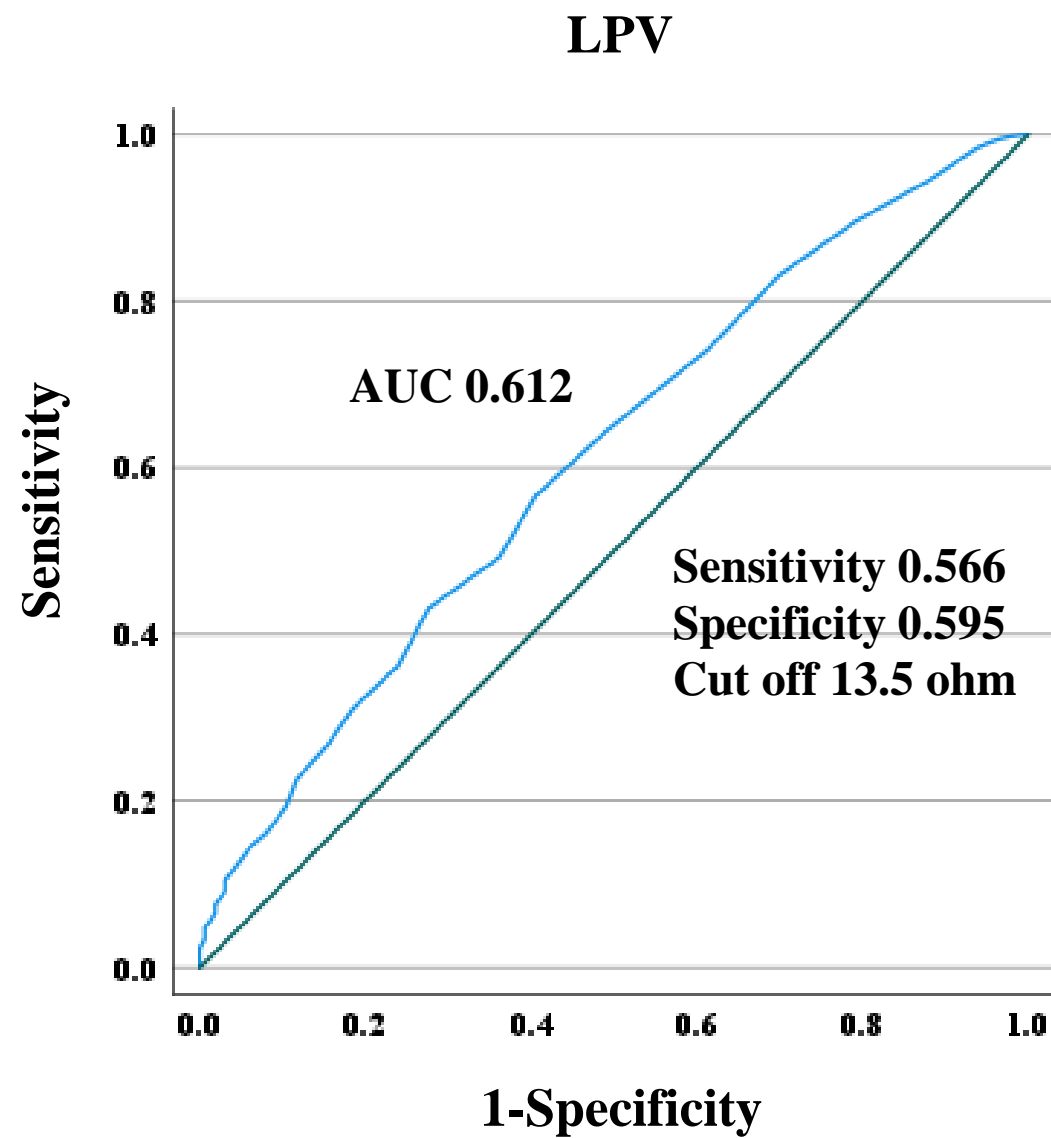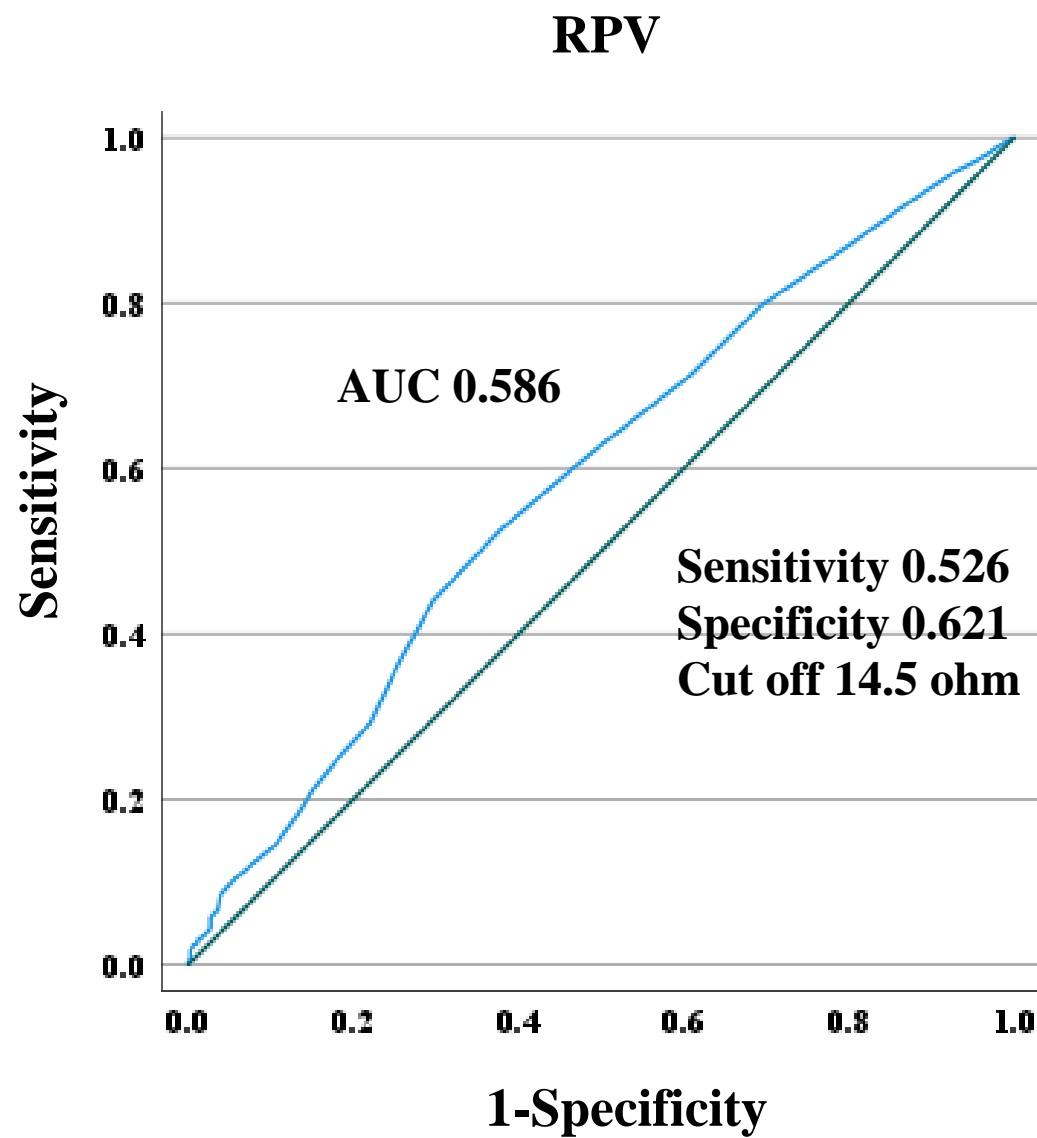

**Supplemental figure 1**

Supplement: Supplementary file 1 — Figure S1. [file JOA3-41-e13191-s001.pdf]
